# A rapid ambient ionization-mass spectrometry approach to monitoring the relative abundance of isomeric glycerophospholipids

**DOI:** 10.1038/srep09243

**Published:** 2015-04-02

**Authors:** Rachel L. Kozlowski, Todd W. Mitchell, Stephen J. Blanksby

**Affiliations:** 1 School of Chemistry, University of Wollongong, Wollongong, NSW, 2522, Australia; 2 Illawarra Health and Medical Research Institute (IHMRI), University of Wollongong, Wollongong, NSW, 2522, Australia; 3 School of Health Sciences, University of Wollongong, Wollongong, NSW, 2522, Australia; 4 Central Analytical Research Facility, Queensland University of Technology, Brisbane QLD, 4001, Australia

## Abstract

Glycerophospholipids with two, non-equivalent fatty acyl chains can adopt one of two isomeric forms depending on the relative position of substitutions on the glycerol backbone. These so-called *sn*-positional isomers can have distinct biophysical and biochemical behaviors making it desirable to uniquely assign their regiochemistries. Unambiguous assignment of such similar molecular structures in complex biological extracts is a significant challenge to current analytical technologies. We have recently reported a novel mass spectrometric method that combines collision- and ozone-induced dissociation in series (CID/OzID) to yield product ions characteristic of acyl chain substitution patterns in glycerophospholipids. Here phosphatidylcholines are examined using the CID/OzID protocol combined with desorption electrospray ionization (DESI) to facilitate the rapid exploration of sample arrays comprised of a wide variety of synthetic and biological sources. Comparison of the spectra acquired from different extracts reveals that the *sn*-positional isomers PC 16:0/18:1 and PC 18:1/16:0 (where the 18:1 chain is present at the *sn-*2 and *sn-*1 position of the glycerol backbone, respectively) are most often found together in lipids of either natural or synthetic origin. Moreover, the proportions of the two isomers vary significantly between extracts from different organisms or even between adjacent tissues from the same organism.

Glycerophospholipids are amphiphilic molecules comprised of a hydrophilic headgroup anchored by a phosphate ester to one of the terminal positions of the glycerol backbone with one or two hydrophobic fatty acids esterified at the remaining hydroxyl positions^1^. This substitution of the glycerol results in chirality about the central carbon with the relative positions of headgroup and acyl chain attachment defined by a *stereospecific numbering* (*sn*) convention^2^. For glycerophospholipids found in eukaryotes, the headgroup is typically esterified at the *sn*-3 position of the glycerol backbone with the fatty acyl chains attached at the *sn*-1 and *sn*-2 positions. While the chirality of glycerophospholipids is thought to be largely conserved across different organisms, for lipids with non-equivalent fatty acyl chains, two regioisomers are possible with alternate substitutions at the *sn*-1 and *sn*-2 positions. Selective enzymatic digests have been used to examine lipid extracts (*vide infra*) and suggest a general trend for unsaturated fatty acyl chains to be preferentially esterified at the *sn*-2 position^3^. There is a growing body of evidence however, that suggests both *sn*-positional isomers are often present and that their proportions may vary in lipid pools of different origin. For example, Figure 1 shows the structures of two *sn*-positional isomers of a phosphatidylcholine substituted with oleic (18:1(9*Z*)) and palmitic (16:0) acids; both of which have been observed in biological extracts^4^,^5^,^6^. Given the potential for distinct biological function(s) for each *sn*-positional isomer, there is a need for analytical methods that can rapidly distinguish these closely related structures and can monitor their relative proportions within complex matrices.

Existing research suggests different biophysical and biochemical properties of glycerophospholipid regioisomers that may confer distinct functions on these isomers in nature. For example, differential scanning calorimetry measurements found significantly different melting temperatures for hydrated bilayers composed of the regioisomers, PC 18:0/16:0 or PC 16:0/18:0, where the longer 18:0 acyl chain is substituted at the *sn*-1 or *sn*-2 position, respectively^7^,^8^. Indeed, this trend was observed between *sn*-positional isomers of many different acyl chain combinations and headgroup classes (including phosphatidylcholines, phosphatidylethanolamines and phosphatidylglycerols) and results from different self-packing efficiencies of the isomers within the bilayer. Recent molecular dynamics simulations of model membrane bilayers incorporating glycerophospholipids and cholesterol reveal important interactions between the off-plane methyl groups of the cholesterol and carbon-carbon double bonds in the acyl chains of the phospholipids. The availability of the double bonds for these preferred interactions is affected by the position of substitution of unsaturated acyl chains on the glycerol backbone such that membrane disorder was found to be greater for PC 16:0/18:1, where the unsaturated acyl chain is located at the *sn*-2 position compared with membranes comprised of the PC 18:1/16:0 isomer^9^. The different susceptibilities of isomeric glycerophospholipids to peroxidation have also been demonstrated. When present in liposomes, 1-palmitoyl-2-linoleoyl-3-*sn*-PC (PC 16:0/18:2) was found to be more sensitive to oxidation by aqueous radicals while 1-linoleoyl-2-palmitoyl-3-*sn*-PC (PC 18:2/16:0) was more susceptible to attack by lipid-soluble radicals^10^.

Different biochemical behaviors of glycerophospholipid *sn*-positional isomers have also been shown *in vivo*. For example, enzymes involved in the modification of glycerophospholipids usually have some degree of specificity for certain acyl chains and/or the site of acyl chain attachment on the glycerol backbone. Human lecithin-cholesterol acyltransferase (LCAT), at first believed to exclusively remove the acyl chain at the *sn*-2 position, has now been shown to have substantial activity for removal of specific acyl chains when present at the *sn*-1 position. Interestingly, while human LCAT utilizes 16:0 acyl chains present at the *sn*-2 position of PC, rat LCAT prefers PC with the 16:0 chain at *sn*-1^11^. Many types of secreted phospholipase A_2_ (PLA_2_) enzymes also have very high specificities for glycerophospholipids containing arachidonic acid (20:4) at the *sn*-2 position^12^. Thus, if both PC 20:4/16:0 and PC 16:0/20:4 are present *in vivo*, PLA_2_ will release arachidonic acid almost exclusively from the latter isomer.

Despite growing evidence of the distinct biochemical and biophysical behaviors of glycerophospholipid *sn*-positional isomers, very little is known about the contribution of lipid isomers to the lipidomes of cells and tissues. This paucity of information arises, in part, from the lack of analytical techniques that are capable of (i) the confident assignment of the positions of acyl chain substitution in glycerophospholipids; and (ii) the rapid assessment of the relative proportions of *sn*-positional isomers within a complex biological sample. Historically, PLA_2_ assays of lipid extracts (or simplified fractions thereof) have used gas chromatography to identify the fatty acids released from the *sn*-2 positions of all glycerophospholipids present^3^,^13^,^14^,^15^. Such approaches provide a global picture of how fatty acyl chains are distributed throughout the lipidome or within a headgroup-class fraction but do not directly identify the substitution pattern(s) at the molecular level.

Modern tandem mass spectrometry approaches enable the rapid identification of the acyl chain composition of a mass-selected lipid ion. This is made possible due to product ions indicative of acyl chain length and degree of unsaturation that are present in the collision-induced dissociation (CID) mass spectra of ionized glycerophospholipids. It has been demonstrated that the relative abundance of these diagnostic ions is often affected by the relative position of substitution on the glycerol backbone and, with care, it can be used to assign the structure of the more abundant *sn*-positional isomer present in an extract. Perhaps the most specific example involves tandem mass spectrometry in negative ion mode where many glycerophospholipids dissociate to yield lysophospholipid product ions from the loss of the acyl chain at the *sn*-2 position^4^,^5^,^16^. Extracting the relative proportions of the *sn*-positional isomers using such methods relies on comparing the abundances of these product ions. Such signals, however, are often of low intensity and their relative abundance can also be affected by differences in lipid structure (*e.g.*, the identity of lipid headgroup and/or fatty acyl chains); the instrument geometry (*e.g.*, ion-trap versus beam-type mass spectrometers); and experimental configuration (*e.g.*, the collision energy applied)^5^,^6^. Such ambiguity in CID data often results in incorrect or over-interpreted reporting of *sn*-position in glycerophospholipids. The identification of this problem has led to new nomenclature for the annotation of lipids based on tandem mass spectra that avoids the assignment of *sn*-position unless it has been explicitly determined (see also Methods - Lipid Nomenclature)^17^.

We have recently introduced an alternative ion activation method that combines collision- with ozone-induced dissociation (OzID)^18^,^19^,^20^. In this strategy, [M+Na]^+^ ions formed from glycerophospholipids during electrospray ionization (ESI) are mass-selected and subjected to CID to remove the headgroup; the resulting product ion is then mass-selected and allowed to react with ozone in an ion trapping region of the mass spectrometer. In contrast to conventional CID approaches, CID/OzID mass spectra show highly abundant product ions that can identify the acyl chain at the *sn*-2 position. Indeed, where both *sn*-positional isomers are present, the relative abundance of the diagnostic CID/OzID ions correlates strongly with the relative proportions of the two lipids^6^. CID/OzID mass spectra can be rapidly acquired and thus the approach can be exploited to survey large numbers of biological samples and to explore isomeric variations between lipid extracts. Conducting such studies using conventional infusion ESI approaches however, runs the risk of contamination by carryover and would thus be rate limited by the time required to purge the system between samples. To circumvent these limitations, we have exploited desorption electrospray ionization (DESI) to enable rapid screening of lipid extract arrays and also examination of lipids directly from tissue sections^21^. Combining DESI with the novel CID/OzID ion activation protocol for the first time, has yielded new insight into the isomeric composition of glycerophospholipids within a diverse range of extracts and also demonstrates how the relative populations of *sn*-positional isomers can vary appreciably, even between adjacent tissues.

## Results

**Figure 2:**
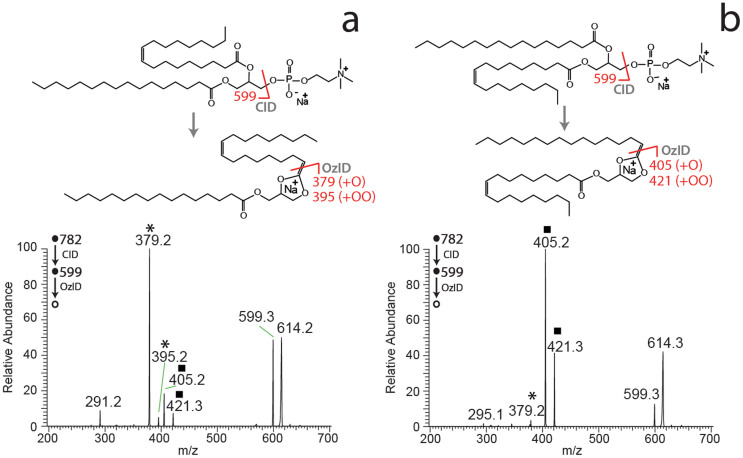
DESI-CID/OzID mass spectra obtained from [M+Na]^+^ ions of synthetic glycerophospholipids (a) PC 16:0/18:1(9*Z*) and (b) PC 18:1(9*Z*)/16:0. The CID/OzID peaks indicative of the presence of the 18:1 chain at the *sn*-2 position (*) and 16:0 at the *sn*-2 position (▪) are marked. The dissociation pathways for the two ionized glycerophospholipid *sn*-positional isomers are indicated above the corresponding mass spectra. Putative dissociation pathways are based on the study of Pham *et al.*^19^ and full reaction mechanisms for CID and OzID steps are provided as Supporting Information (Scheme S1). Spectra acquired using offline ozone generation (see Methods).

The ability of the DESI-CID/OzID method to distinguish *sn*-positional isomers of glycerophospholipids was examined using a pair of synthetic phosphatidylcholine standards namely, PC 16:0/18:1(9*Z*) and PC 18:1(9*Z*)/16:0. Each compound was prepared in acetonitrile/2-propanol and the solutions were loaded onto arrays of PTFE spots on glass sample slides before being dried and subjected to DESI analysis. Abundant [M+Na]^+^ ions were observed from the lipids deposited on each spot and these ions were then subjected to CID in the ion-trap mass spectrometer to yield the abundant [M+Na-183]^+^ product ion resulting from neutral loss of the phosphocholine headgroup^22^,^23^. This product ion was then re-isolated and retained in the ion trap in the presence of ozone for 200 ms before the fragment ions were mass analyzed giving the mass spectra shown in Figure 2.

The DESI-CID/OzID mass spectra obtained for PC 16:0/18:1(9*Z*) and PC 18:1(9*Z*)/16:0 are significantly different with the former dominated by a base peak of *m/z* 379 with a low abundant product ion at *m/z* 405 (Figure 2a), while the latter has a base peak at *m/z* 405 with the signal at *m/z* 379 barely visible above the noise (Figure 2b). Previous studies identify the ion at *m/z* 379, and its companion peak at *m/z* 395, as resulting from oxidative cleavage of the 18:1 acyl chain from the *sn*-2 position of the glycerol backbone. Conversely, the *m/z* 405 and its companion peak at m/z 421 are markers for the presence of the 16:0 acyl chain at *sn*-2. The CID/OzID reaction pathways giving rise to these diagnostic ions have previously been proposed^19^ and are summarized above the relevant spectra in Figure 2. Full reaction schemes, based on current understanding of the reaction mechanism, are provided as Supporting Information (see Scheme S1). Importantly, these CID/OzID product ions can thus be used to identify the isomeric composition of the lipids present on the surface. The presence of the *m/z* 405, 421 ion pair in the spectrum obtained from PC 16:0/18:1(9*Z*) (Figure 2a) and conversely the presence of *m/z* 379 in the spectrum obtained from PC 18:1/16:0(9*Z*) (Figure 2b) suggest that neither sample is isomerically pure. This observation is consistent with previous mass spectrometric and enzymatic analyses of synthetic glycerophospholipids, that find some abundance of the alternative regioisomer is nearly always present^5^,^6^. This is likely a result of acyl chain migration during synthesis procedures^24^.

DESI-CID/OzID mass spectra obtained from both synthetic PC isomers show a broad peak centered on *m/z* 614 (Figure 2). This feature has previously been suggested to be a fragile epoxide of actual *m/z* 615 that dissociates upon mass analysis in the ion trap resulting in an unusual broad peak shape and a lower apparent *m/z* ratio^19^. Putative structures for these ions, based on prior investigations, are provided as Supporting Information (see Scheme S1) and their appearance as broad, tailing peaks of apparently lower mass is consistent with the well-documented behavior of fragile ions in ion-trap mass spectrometers^25^,^26^. Another interesting observation from the data presented in Figure 2 is that the overall conversion of the mass-selected *m/z* 599 into product ions is lower for PC 16:0/18:1(9*Z*) (Figure 2a) than for PC 18:1(9*Z*)/16:0 (Figure 2b). Given that the time permitted for reaction of the ions at *m/z* 599 with ozone (200 ms) is the same for each isomer, this observation suggests slightly different rates of reaction for the isomeric ions undergoing ozonolysis in each case. As a consequence, for the concentration of ozone and the reaction time employed in this study, a slight detection bias exists in favor of PC 18:1(9*Z*)/16:0 over PC 16:0/18:1(9*Z*). This small bias could be removed by extending reaction times such that *m/z* 599 was completely quenched but this would consequently increase the overall analysis time. Comparison to prior measurements of these standards using alternative methods (see later) suggested that small bias was acceptable given that the aim of this investigation was to develop a method to rapidly probe changes in relative *sn*-positional isomer populations for comparison between extracts rather than to obtain absolute concentrations for each lipid.

**Figure 3:**
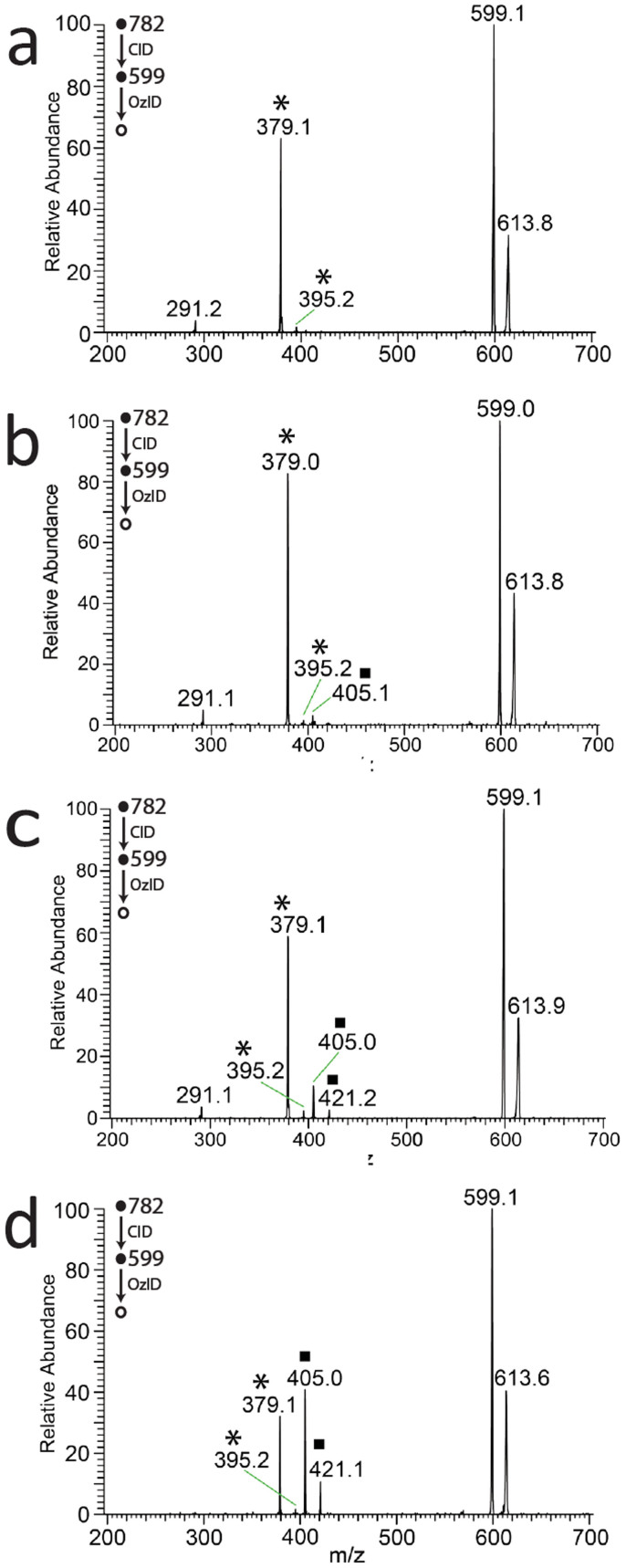
DESI-CID/OzID mass spectra obtained from the [M+Na]^+^ ions at *m/z* 782 corresponding to PC 34:1 isomers from (a) egg yolk, (b) cow perinephric adipose, (c) cow kidney medulla and (d) cow ocular lens. Peaks indicative of the *sn*-positional isomers PC 16:0/18:1 (*) and PC 18:1/16:0 (▪) are marked as indicated. Spectra acquired using online ozone generation (see Methods).

Once optimized for synthetic glycerophospholipids, DESI-CID/OzID analysis was undertaken for lipid extracts obtained from a range of biological sources. Representative mass spectra obtained from *m/z* 782 produced upon DESI of deposited extracts from egg yolk, cow kidney and cow eye lens are shown in Figure 3. In all cases the spectra are consistent with the [M+Na]^+^ ions formed from the abundant monounsaturated phosphatidylcholine, PC 34:1 and show product ions identical to those observed for the PC 16:0_18:1 isomers shown in Figure 2. Notably, the relative abundance of product ions compared to *m/z* 599 in the spectra in Figure 3 is lower than that shown in Figure 2. This reflects the different ozone concentration present in the ion trap when each data set was acquired. Significantly however, the ozone concentration was constant during the acquisition of all spectra in Figure 3. As such, the changes in relative abundance of the diagnostic product ions pairs at *m/z* 379, 395 and *m/z* 405, 421 are evidence of different proportions of the *sn*-positional isomers PC 16:0/18:1 and PC 18:1/16:0 in each of the lipid extracts. Even a qualitative comparison of the differences between the spectra shown in Figure 3 suggests significant variation in isomeric composition between samples. For example, in the CID/OzID spectrum from egg yolk (Figure 3a) the ion pair at *m/z* 379, 395 is dominant with the very little signal for *m/z* 405, 421 detectable above the noise. This suggests that phosphatidylcholines of the form PC 34:1 present in egg yolk comprise almost entirely PC 16:0/18:1. Indeed, the very low abundance of product ions at *m/z* 405, 421 suggests that there is very little of the regioisomer PC 18:1/16:0 present in egg yolk - even less than the isomeric impurity observed for the PC 16:0/18:1 synthetic standard (*cf*. Figure 2a). Comparison of the analogous spectra obtained from extracts of cow perinephric adipose tissue and kidney medulla (Figures 3b and c) shows the ion pair at *m/z* 379, 395 again dominating the product ion signals but with *m/z* 405, 421 readily observable. These data suggest that PC 16:0/18:1 is still the major isomer in these tissues but - unlike egg yolk - significant amounts of PC 18:1/16:0 are also present. Intriguingly, variation in the relative abundance of the diagnostic product ions is also apparent when comparing spectra obtained from different regions of the same tissue (*cf*. Figures 3b and c). Finally, the CID/OzID spectrum obtained from a cow ocular lens extract shows product ion pairs at *m/z* 379, 395 and *m/z* 405, 421 to be of comparable abundance. This suggests that the isomers PC 16:0/18:1 and PC 18:1/16:0 are likely to be present in near equal abundance in this tissue.

**Figure 4:**
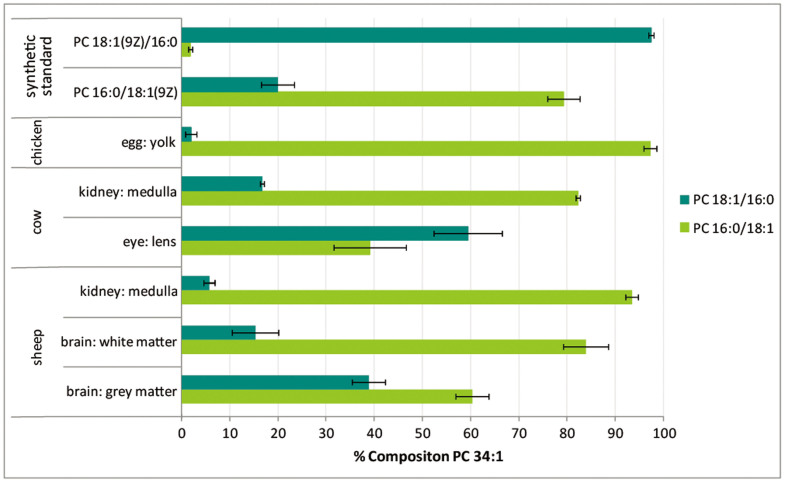
The estimated abundance of *sn*-positional isomers PC 18:1/16:0 and PC 16:0/18:1 as a percentage of phosphatidylcholines of composition PC 34:1 in different biological extracts and commercially available synthetic standards. Isomer contributions are estimated from the normalized product ion abundances in DESI-CID/OzID mass spectra as described in the text. Product ions assigned to isomers of PC 16:1_18:0 were included in all computations but represented < 2% of the composition and are thus not shown here. All data points represent an average of at least four technical and, where available, biological replicates (see Supporting Information Table S1). Uncertainties shown represent standard deviation. This graph was generated using spectra acquired using offline and online ozone generation methods (see Methods).

The DESI interface enabled the rapid acquisition of CID/OzID mass spectra from an array of lipid extracts spotted onto sample slides (see Methods). All DESI-CID/OzID mass spectra of *m/z* 782 ions were dominated by the product ion pairs at *m/z* 379, 395 and *m/z* 405, 421 indicative of the *sn*-positional isomers PC 16:0/18:1 and PC 18:1/16:0, respectively. In some instances, low abundant product ion pairs were also observed at *m/z* 377, 393 and *m/z* 407, 423 which, by analogy to the analysis above, were assigned to the *sn*-positional isomers PC 16:1/18:0 and PC 18:0/16:1, respectively. From the spectra acquired at each spot on the array, the relative contribution of a single isomer to the PC 34:1 lipid population could be estimated by normalizing the abundance (*A_i_*) of the characteristic ion pair for the nominated isomer (*e.g.*, the combined ion abundance [*A_379_*+*A_395_*] of *m/z* 379 and 395 formed from PC 16:0/18:1 as indicated in Equation 1) to the sum of product ion signals from all four contributors. Combining these data across replicate measurements enabled a more rigorous comparison of changes in isomer profiles between lipid extracts of different biological origins and the results are summarized in Figure 4. Estimates of the relative contribution of the PC 16:1/18:0 and PC 18:0/16:1 to the composition PC 34:1 were found to be typically less than 2% (see Supporting Information Table S1) and are thus not presented in Figure 4.
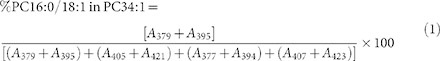
The estimates of relative contribution shown in Figure 4 indicate that the synthetic samples supplied as PC 16:0/18:1(9*Z*) and PC 18:1(9*Z*)/16:0 include levels of the alternate isomer at *ca*. 20% and 2%, respectively. These results are consistent with recent investigations of synthetic standards using CID/OzID with infusion electrospray, ion-mobility separations and enzymatic assays (see Supporting Information Table S2 for comparison)^6^,^19^. In contrast to the synthetic standard, PC 34:1 in egg yolk was found to be almost exclusively comprised of a single *sn*-positional isomer. With an estimated abundance of 97% PC 16:0/18:1 this extract was found to be the most isomerically pure of any biological sample investigated in this study. Cow kidney tissue (medulla) was found to have both isomers present with PC 16:0/18:1 and PC 18:1/16:0 estimated to comprise 82% and 17%, respectively, of the PC 34:1 population. Interestingly, the analogous tissue in sheep showed a relative amount of the PC 18:1/16:0 isomer of only 6%, while sheep brain tissue had the same isomer present at 15% in white matter and 39% in grey matter. Finally, the estimates in Figure 4 confirm the preliminary finding that the PC 18:1/16:0 isomer is likely more abundant than PC 16:0/18:1 in lipid extracts of cow eye lens. While these data do not provide absolute quantification of the isomers present in these extracts, they provide a unique insight into the substantial variation in the relative abundance of these two distinct lipid molecules in samples of different biological origin (Figure 4).

**Figure 5:**
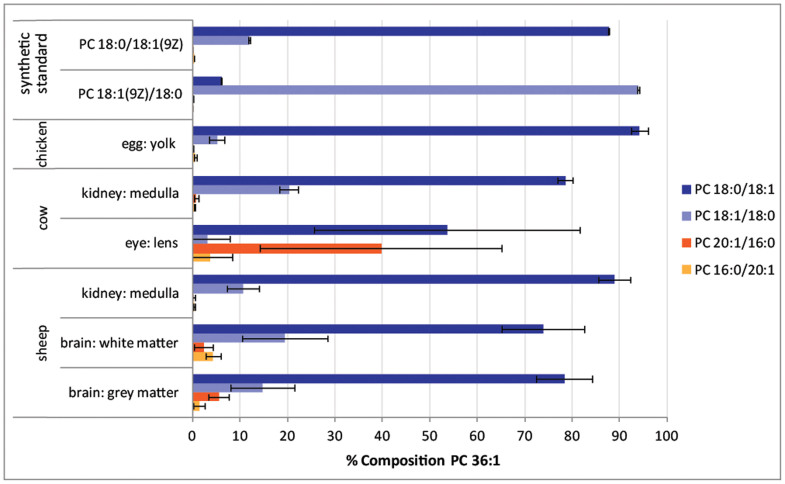
The estimated abundance of *sn*-positional isomers PC 18:0/18:1; PC 18:1/18:0; PC 20:1/16:0; and PC 16:0/20:1 as a percentage of phosphatidylcholines of composition PC 34:1 in different biological extracts and commercially available synthetic standards. Isomer contributions are estimated from the normalized product ion abundances in DESI-CID/OzID mass spectra as described in the text. All data points represent an average of at least four technical and, where available, biological replicates (see Supporting Information Table S1). Uncertainties shown represent standard deviation. This graph was generated using spectra acquired using offline and online ozone generation methods (see Methods).

The data summarized in Figure 4 suggest wide variation in isomer contributions to the PC 34:1 lipid population. In an effort to establish if this was unique to phosphatidylcholines of this composition, the isomeric contributions to PC 36:1 were also investigated. The same sample arrays were examined by using sequential CID of mass-selected [M+Na]^+^ precursor ion at *m/z* 810 and subjecting the abundant product ion [M+Na-183]^+^ at *m/z* 627 to OzID. Thus obtained, the CID/OzID spectra yielded a series of product ion pairs that could be assigned to the isomers indicated: *m/z* 379, 395 corresponding to PC 16:0/20:1; *m/z* 433, 499 corresponding to PC 20:1/16:0; *m/z* 407, 423 corresponding to PC 18:0/18:1 and; *m/z* 405, 421 corresponding to PC 18:1/18:0. Comparing the relative peak areas of these diagnostic ions according to an analogous version of Equation 1 enabled an estimate of the relative contribution of these isomers to the total PC 36:1 pool. These data are provided as supporting information (Table S1) and are summarized in Figure 5.

The data presented in Figure 5 suggest that commercially available synthetic standards PC 18:0/18:1(9*Z*) and PC 18:1(9*Z*)/18:0 contain detectable contributions from the alternate *sn*-positional isomer at *ca*. 12% and 6%, respectively. This is comparable to the data obtained from synthetic lipid PC 16:0/18:1(9*Z*) and PC 18:1(9*Z*)/16:0 (see Figure 4). Interestingly however, the PC 36:1 pool of lipids in egg yolk shows the presence of both PC 18:0/18:1 at ~94% and PC 18:1/18:0 at ~5% whereas the PC 34:1 lipids in the same extract are comprised almost exclusively of PC 16:0/18:1. For the remaining biological extracts the lower abundance of PC 36:1 compared to PC 34:1 led to lower signal-to-noise in the DESI-CID/OzID spectra obtained from the former. As a result, technical variation was larger in these data; as evidenced by the more substantial standard deviations shown in Figure 5. Notwithstanding this variation, the overall trend is of similar abundance ratios of PC 18:0/18:1 to PC 18:1/18:0, with the former being substantially more abundant in every case. These findings contrast with the widely varying ratios of PC 16:0/18:1 to PC 18:1/16:0 reported in Figure 4. In a similar way, DESI-CID/OzID analysis was undertaken for lipids of composition PC 34:2 and PC 36:2. The lower abundance of these lipids meant that reliable data could not be obtained across the full sample set and these results are summarized as Supporting Information (see Table S1).

**Figure 6:**
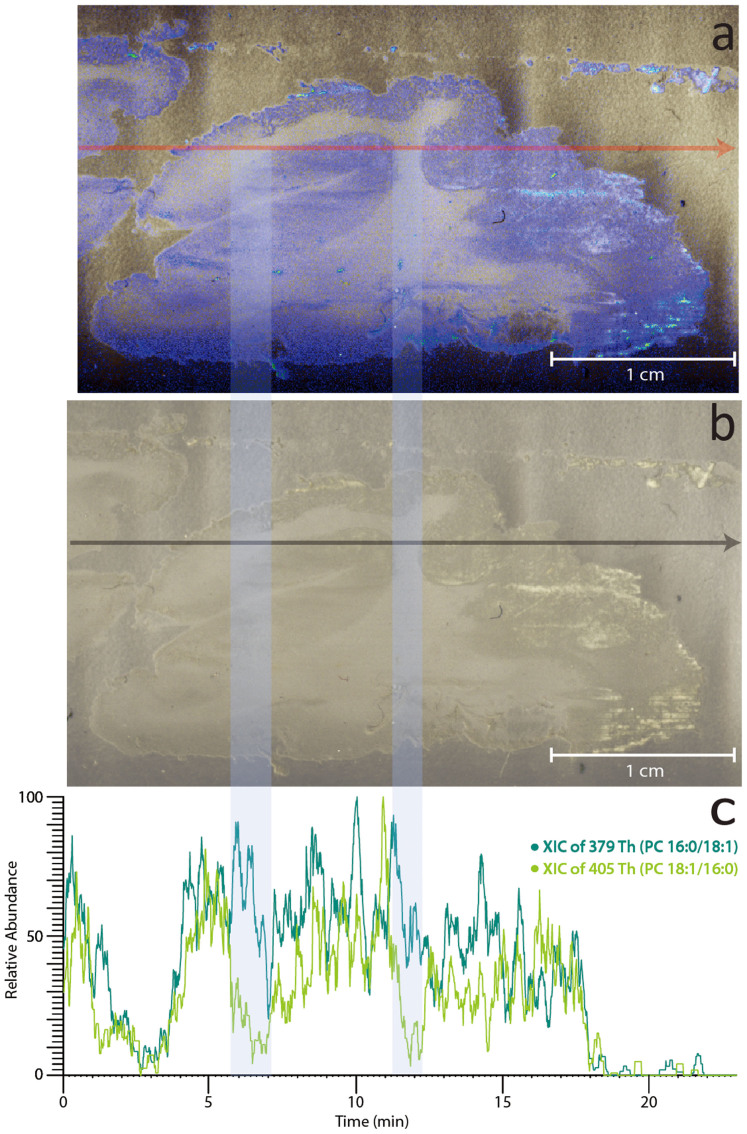
DESI-CID/OzID analysis of isomers of PC 34:1 desorbed directly from a sheep brain tissue section. Panels (a) and (b) show a photograph of the sheep brain tissue section analyzed with panel (a) digitally colored to highlight the regions of grey matter (blue) and white matter (white). (c) The overlaid extracted ion chronograms acquired from a single transect (indicated by the grey arrow in the panel b) for diagnostic ions at *m/z* 379 (dark green) corresponding to PC 16:0/18:1, and *m/z* 405 (light green) corresponding to PC 18:1/16:0. The light blue shading indicates where the transect crosses regions of white matter.

The observation that adjacent tissue extracts had variation in the relative abundance of *sn*-positional isomers PC 16:0/18:1 and PC 18:1/16:0 (*cf*. grey and white matter extracts in Figure 4) suggested that it may be possible to visualise the changing isomer populations from direct analysis of the tissue itself. To examine this, a thin section of sheep brain tissue was prepared, mounted on a glass slide and subjected to DESI-CID/OzID. The data acquired from direct analysis of sheep brain tissue are summarised in Figure 6 and show ion chronograms obtained from the single transect of the tissue under the DESI emitter. The extracted ion chronograms of the diagnostic *m/z* 379 and *m/z* 405 product ions plotted in Figure 6 can be considered to represent the abundance of PC 16:0/18:1 and PC 18:1/16:0, respectively. These data show that the abundance of the m/z 379 ion remains relatively constant across the tissue boundaries. In contrast, a substantial drop in the relative abundance of the *m/z* 405 ion is observed in white matter compared to grey (see shaded areas in Figure 6). This finding suggests that while PC 16:0/18:1 is somewhat evenly distributed throughout the brain, PC 18:1/16:0 is differentially distributed between grey and white matter. These data represent an important proof-of-principle that this approach can be used to visualise changes in lipid isomer populations in direct tissue analysis. Similar transects were repeated across the entire tissue and the abundance data were reconstructed as mass spectral images. These images are provided as Supporting Information (Figure S4) but the low signal-to-noise in these data made resolving the different brain regions challenging. Nevertheless, there is some delineation between white and grey matter in the image corresponding to the distribution of PC 18:1/16:0 (Figure S4b).

## Discussion

In this study, we have demonstrated the utlility of DESI-MS in analyzing arrays of lipid extracts. No cross-contamination between adjacent samples was noted as evidenced by the data obtained from egg yolk showing a near pure sample of the PC 16:0/18:1 isomer. DESI-MS has proven to be an extremely useful means to rapidly survey the phosphatidylcholines present in a diverse range of lipid extracts. Combining DESI with ion activation by CID/OzID, for the first time, has enabled the acquisition of spectra that uniquely identify the presence of each of the possible *sn*-positional isomers of asymmetric diacylphosphatidylcholines. Such clear differentiation of isomeric lipids would not be possible using conventional tandem mass conventional tandem mass spectra. By comparing the relative abundance of diagnostic marker ions it has been shown that the relative contribution of each isomer to the lipid population can be determined. While only providing an approximation, this approach has enabled the comparison of the relative isomer populations in biological extracts and has revealed remarkable variation in the proportions of *sn*-positional isomers in nature. Data obtained for the isomeric pair, PC 16:0/18:1 and PC 18:1/16:0, suggest that while common assumptions that the unsaturated acyl chain is found at the *sn*-2 position are borne out in some biological extracts (*e.g*., egg yolk), such assumptions would lead to the wrong conclusions in others (*e.g.*, the near equal proportions of the two possible isomers found in cow ocular lens). Although phosphatidylcholines were the only lipid class investigated here, in previous infusion ESI studies CID/OzID has been proven effective for examining *sn*-positional isomers of all major glycerophospholipid classes and even some triacylglycerols^19^,^20^. This suggests that the methods described here could be deployed in the future to examine the similarlities and differences in acyl chain substitution patterns across the glycerolipidome. Such data could help in understanding the biosynthetic connections between different lipid structures.

The results of the current study serve to reinforce the importance of careful annotation of lipid mass spectral data to ensure lipid structures are not incorrectly assigned to a single *sn*-positional isomer based only on CID data. Based on our findings it seems reasonable to project that in most living systems both isomers are present albeit in different proportions. The underlying reasons for these subtle, molecular-level differences between lipidomes remains to be determined. It is clear however, that understanding the biological roles of different lipid isomers requires analytical tools such as those described here.

Direct analysis of sheep brain tissue by DESI-CID/OzID has revealed that while PC 34:1 is homogeneously distributed throughout, the relative concentration of the constituent isomers has local variation (*cf*. Figure 6 and Supporting Information Figure S4). These data highlight current limitations in imaging mass spectrometry for lipid applications where monitoring pseudo-molecular ions (enabling assignment at the sum composition level, *e.g.*, PC 34:1) or even CID product ions (enabling assignment of the acyl chain composition level, *e.g.*, PC 16:0_18:1) may not be sufficient to reveal regional differences in lipid abundances. As such, further development of targeted ion activation strategies is required, to be used in combination with desorption/ionization mass spectrometry to enable more precise assignment of lipid molecular structure and distribution. These protocols must yield abundant and diagnostic product ions for closely related lipid isomers and must be obtained on short timescales and on relatively low abundant ions. The data presented here suggest that, with further optimization, CID/OzID may serve as one such method for future applications.

## Methods

### Materials

All solvents used, including water, were Optima LCMS grade (Thermo Fisher Scientific, Scoresby, VIC, Australia). Butylated hydroxytoluene (BHT) was purchased from Sigma-Aldrich (St. Louis, MO, USA). Ammonium chloride and sodium acetate were analytical grade and purchased from Ajax Chemicals (Auburn, NSW, Australia). Industrial-grade compressed oxygen was purchased from BOC (Cringila, NSW, Australia). Synthetic glycerophospholipid standards PC 18:1(9*Z*)/18:1(9*Z*) (1,2-di-(9*Z*-octadecenoyl)-*sn*-glycero-3-phosphocholine); PC 16:0/18:1(9Z) (1-hexadecanoyl-2-(9Z-octadecenoyl)-*sn*-glycero-3-phosphocholine); PC 18:1(9*Z*)/16:0 (1-(9*Z*-octadecenoyl)-2-hexadecanoyl-*sn*-glycero-3-phosphocholine); PC 18:0/18:1(9*Z*) (1-octadecanoyl-2-(9Z-octadecenoyl)-*sn*-glycero-3-phosphocholine); and PC 18:1(9*Z*)/18:0 (1-(9Z-octadecenoyl)-2-octadecanoyl-*sn*-glycero-3-phosphocholine) were purchased from Avanti Polar Lipids, Inc. (Alabaster, AL, USA). *L*-α-Phosphatidylcholine from egg yolk was purchased from Sigma-Aldrich (St. Louis, MO, USA) and all biological tissues used for extractions and tissue sections were purchased from Kieraville Butchery (Kieraville, NSW, Australia). Where available, biological replicates were obtained and these details are provided as Supporting Information (see footnote to Table S1).

### Lipid Nomenclature

Lipid nomenclature used here is guided by the recommendations of the Lipid MAPS consortium and the recent suggestions of Liebisch *et al*. for mass spectrometry derived data^17^,^27^. Briefly, the number of carbon atoms present in an acyl chain is written before the colon and the number of double bonds after the colon, such that “18:1” represents an acyl chain containing 18 carbon atoms and 1 double bond. If the double bond positions are known, these are noted in brackets after the acyl chain double bond number, as counted from the carboxyl carbon of the fatty acid, *e.g.*, 18:1(9). If the *E*/*Z* configurations of the double bonds are known, they are noted within brackets immediately after each corresponding double bond position, *e.g.*, 18:1(9*Z*). For glycerophospholipids, the headgroup class is indicated by the two letter abbreviation, *e.g.*, phosphatidylcholine is presented as PC and the acyl chains at the (*stereospecifically numbered*) *sn*-1 and *sn*-2 positions are presented before and after the forward slash, respectively (*e.g.*, PC 16:0/18:1 indicates 16:0 at the *sn*-1 position and 18:1 at the *sn*-2 position). Where the acyl chain composition is known but the relative positions of the chains on the glycerol backbone are uncertain, or a mixture of isomers may be present, the acyl chains are separated by an underscore, *e.g.*, PC 16:0_18:1.

### Sample Preparation

The synthetic glycerophospholipid standards, PC 16:0/18:1(9*Z*), PC 18:1(9*Z*)/16:0, PC 18:1(9*Z*)/18:1(9*Z*), PC 18:0/18:1(9*Z*) and PC 18:1(9*Z*)/18:0, were each separately dissolved to a final concentration of 200 µM in acetonitrile/2-propanol (50/50, v/v). Egg yolk extract was weighed then dissolved in acetonitrile/2-propanol (50/50, v/v) to an approximate final concentration of 175 µM total lipid. For biological tissues 15–150 mg of tissue was dissected and extracted using a modified Folch extraction method^28^,^29^. Briefly, dissected tissues were homogenized using a bead homogenizer (glass beads, 1 mm diameter) and extracted using methanol/chloroform (33/67 v/v). The organic extract was washed three times with water, and once with aqueous ammonium chloride (50 mM) before being dried under a stream of nitrogen and redissolved with 750 µL of acetonitrile/2-propanol (50/50, v/v). Aliquots (3 µL) of the lipid solutions were deposited onto PTFE sample spots on dedicated glass slides (Prosolia, Indianapolis, IN, USA) for DESI analysis. Technical replicates of each standard or extract were prepared, with each solution applied to a minimum of four PTFE spots that were rapidly air dried (*ca*. 5 mins) and immediately stored. Sample arrays were stored in an airtight box at −80°C to avoid unwanted oxidation by ambient ozone (see also Supporting Information, Figure S3)^30^,^31^.

For direct tissue analysis, one hemisphere of sheep brain cerebrum was flash frozen at −80°C and coronal slices were sectioned to a thickness of 30 µm using a Leica cryostat CM1950 (Leica, St Louis, MO, USA) maintained at −17°C. The sheep brain section was then mounted onto a glass slide and stored at −20°C until analysis.

### Mass Spectrometry

Mass spectra were acquired using an linear ion-trap mass spectrometer operating Xcalibur 2.0 control software (LTQ Thermo Fisher Scientific, San Jose, CA, USA). The instrument has been previously modified to undertake ion-molecule reactions^32^. Ions were generated using a desorption electrospray ionization source fitted with a motorised DESI 2D Source (Prosolia, Indianapolis IN, USA). The LTQ heated capilary inlet was replaced with a modified inlet capillary (Prosolia, Indianapolis IN, USA). The source was configured with the spray emitter tip angled at 55° with respect to the plate, while the height of the emitter tip and the sample stage were both optimized with respect to the inlet to maximize signal intensity. The stage holding the slide was set at 9 mm in the z-axis while the emitter tip was set at 11 mm in the x-axis, 6 mm in the y-axis and 17 mm in z-axis as determined by the markings on the DESI source. A mixture of acetonitrile and isopropoanol (50/50 v/v) containing sodium acetate (100 μM) was used as the spray solvent at a flow rate of 7 µL/min for analyses of biological extracts and lipid standards and 2 µL/min when sampling directly from tissue. For long acquisitions, a liquid chromatography pump was used to provide a constant flow rate for DESI experiments (Surveyor HPLC system, Thermo Fisher Scientific, San Jose, CA). The DESI sample stage was traversed at rates of 50–100 µm/s for analyses of biological extracts and lipid standards and 25 µm/s for tissue sections. Mass spectrometer parameters used were as follows: ionspray voltage 5.00 kV; capillary temperature 275°C; the capillary voltage −35.0 V; and tube lens 125.0 V. Automatic gain control was turned on (as is the default) but ion counts were typically low and thus the instrument always operated with the maximum ion injection time of 500 ms. Under these conditions limits of detection for a single phosphtaidylcholine on a PTFE spot were estimated at 40 fmol. CID/OzID spectra were obtained by operating the mass spectrometer in MS^3^ mode with ozone present in the ion trapping region. CID of the mass-selected [M+Na]^+^ precursor ion (isolation width 5 Th) was performed with a normalized collision energy of 38 (arbitrary units) and an activation time of 30 ms. The targeted [M+Na-183]^+^ product ion was then isolated (isolation width 3 Th) and trapped for 200 ms in the presence of ozone before mass-selective ejection of all ions from the trap. Experimental parameters for ozone-induced dissociation on this platform have been previously described and involve the introduction of ozone generated off-line and collected in a plastic syringe^33^,^34^. Recent modifications to this procedure enable online generation and supply of ozone (see Supporting Information, Figure S1 for a schematic of the instrument configuration), providing a stable concentration of ozone over extended periods^35^,^36^. Both methods have been used to obtain the CID/OzID spectra reported herein and these are indicated throughout as “offline” and “online”, respectively (see also Supporting Information Figure S2 and associated discussion). Spectra used in this study represented an average of *ca*. 30–70 individual scans.

## Author Contributions

R.L.K., T.W.M. and S.J.B. designed the project. R.L.K. conducted all experiments. R.L.K. and S.J.B. wrote the main manuscript text and prepared figures. All authors reviewed the manuscript and supporting information.

## Supplementary Material

Supplementary Information Supplementary Information

## Figures and Tables

**Figure 1:**
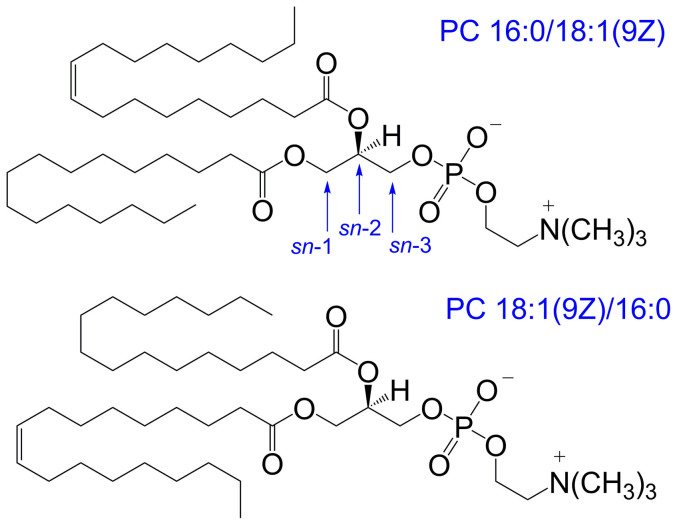
Structures of the phosphatidylcholine *sn*-positional isomers, PC 16:0/18:1(9Z) and PC 18:1(9Z)/16:0 that differ only in the relative position of the acyl chains on the glycerol backbone. The stereospecific numbering (*sn*) of carbons of the glycerol backbone is indicated.
