# Supplementary material for: A rapid ambient ionization-mass spectrometry approach to monitoring the relative abundance of isomeric glycerophospholipids
Source: Sci Rep. 2015 Apr 2;5:9243. doi: 10.1038/srep09243 (PMC4399504; doi:10.1038/srep09243)
Supplement: Supplementary Information [file srep09243-s1.pdf]

Supporting Information for:

A rapid ambient ionization-mass spectrometry approach to monitoring the relative abundance of isomeric glycerophospholipids

Rachel L. Kozlowski[a,b], Todd W. Mitchell*[b,c] and Stephen J. Blanksby*[a,d]

[a] *School of Chemistry, University of Wollongong, Wollongong, NSW, 2522, Australia*

[b] *Illawarra Health and Medical Research Institute (IHMRI), University of Wollongong, Wollongong, NSW, 2522, Australia*

[c] *School of Health Sciences, University of Wollongong, Wollongong, NSW, 2522, Australia*

[d] *Central Analytical Research Facility, Queensland University of Technology, Brisbane QLD, 4001, Australia*

*** Authors to whom correspondence should be addressed**

stephen.blanksby@qut.edu.au

todd_mitchell@uow.edu.au

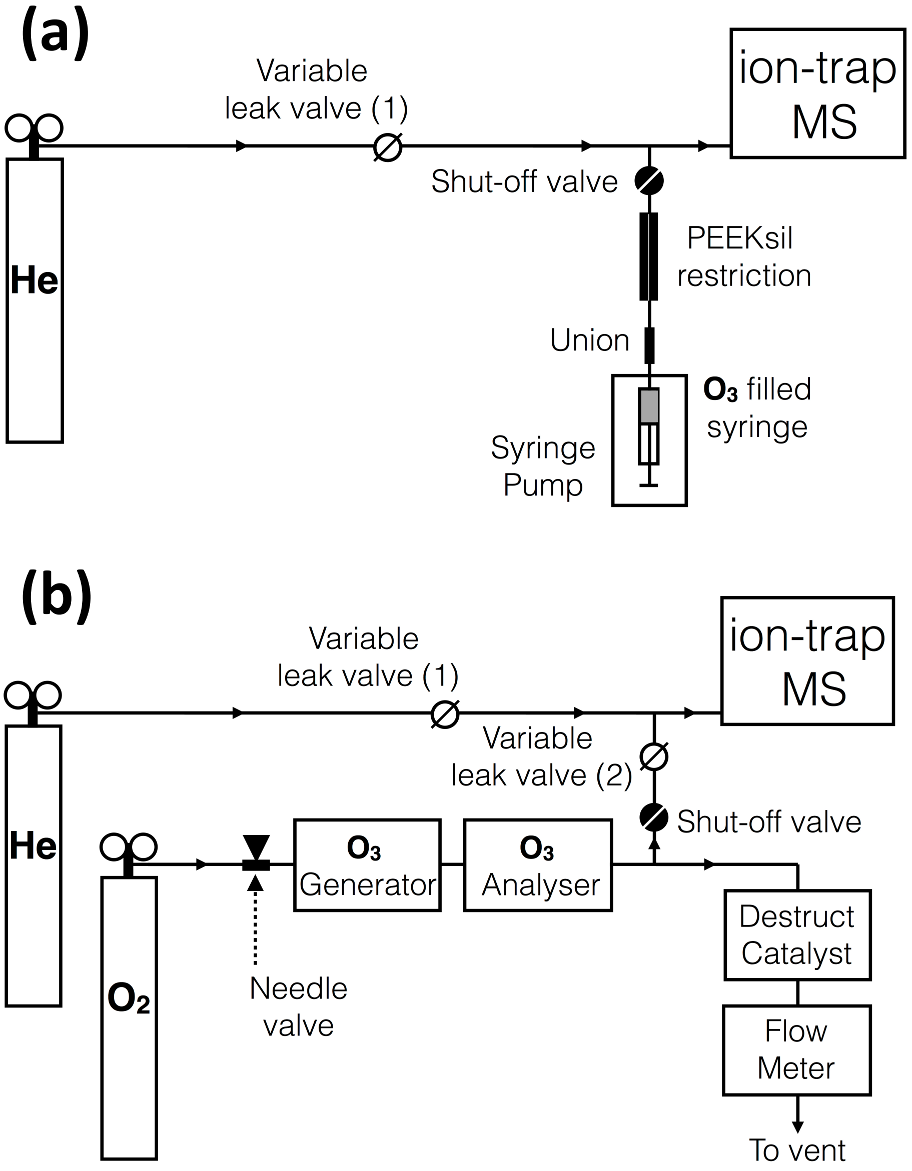

Figure S1.

(a) Schematic illustrating the *offline* setup for delivery of ozone to a modified linear ion-trap mass spectrometer (LTQ Thermo Fisher Scientific, San Jose CA, USA). Ozone is generated offline using a HC-30 ozone generator (Ozone Solutions, Sioux Center, IA, USA) and collected in a suitable gas tight plastic syringe. Gas from the syringe is delivered to the helium buffer gas via a PEEKsil restriction (100 mm L × 1/16 in. OD × 0.025 mm ID, SGE Analytical Science, Ringwood, Vic, Australia). This method, including the safe generation and collection of ozone, is described in detail in reference[1].

(b) Schematic illustrating the *online* ozone generation and delivery setup for the linear ion-trap mass spectrometer. Ozone is generated using a 0.2 L min^{-1} flow of industrial grade oxygen through an ozone generator (Titan 100 generator, Absolute Ozone, Edmonton Canada) and was measured at *ca*. 170 g m^{-3} (normal) using and inline ozone analyzer (Mini HiCon; InUSA Inc., Norwood, MA, USA). Thus formed, a low flow of ozone/oxygen was added to the ion trap supply helium buffer gas using a variable leak valve (VSE Vacuum, Lustenau, Austria). This method is based on previous developments for online ozone generation and delivery for ion-trap mass spectrometers as described in reference[2].

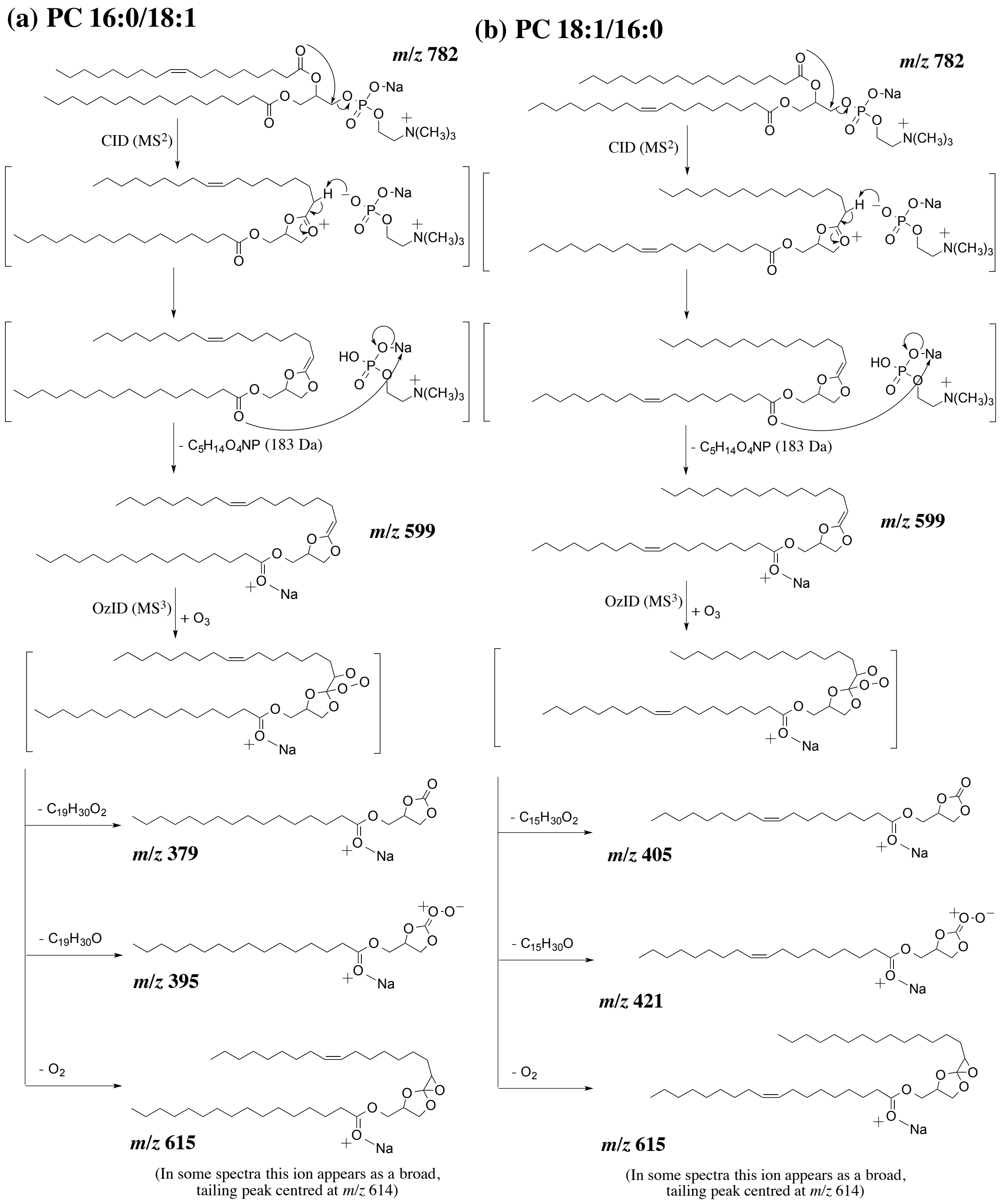

Scheme S1. Proposed mechanism to account for the CID/OzID product ions observed in the spectra shown in Figures 2 and 3 from **(a)** $[PC\ 16:0/18:1 + Na]^+$ and **(b)** $[PC\ 18:1/16:0 + Na]^+$. Mechanisms and product ion structures are based on proposals outlined in reference[3].

Day-to-day variation in abundance data when using offline ozone generation

Figure S2 displays the day-to-day variation for DESI-CID/OzID measurements of synthetic PC 18:1(9Z)/16:0 and PC 16:0/18:1(9Z), and a phosphatidylcholine extract from chicken egg yolk. Although the values for the % composition for analogous *sn*-positional isomers does not appear to vary from day to day, for all 3 samples, the values obtained on day 1 fall outside the error bars of those obtained on the second day. These data were acquired using "offline" ozone generation and so the variability observed likely arises from different ozone concentrations in the ion trap for the experiments conducted on different days. As noted in the main body of the manuscript, the two *sn*-positional isomers have slightly different rates of reaction at the OzID step in the sequence thus with different ozone concentrations the abundance of characteristic ions can vary accounting for the observed day-to-day variability in these data. Generating ozone "online" has been shown to provide reproducible ozone concentrations.[2,4] Thus the experimental configuration was modified as shown in Figure S1 to provide a continuous, stable supply of ozone to the ion trap.

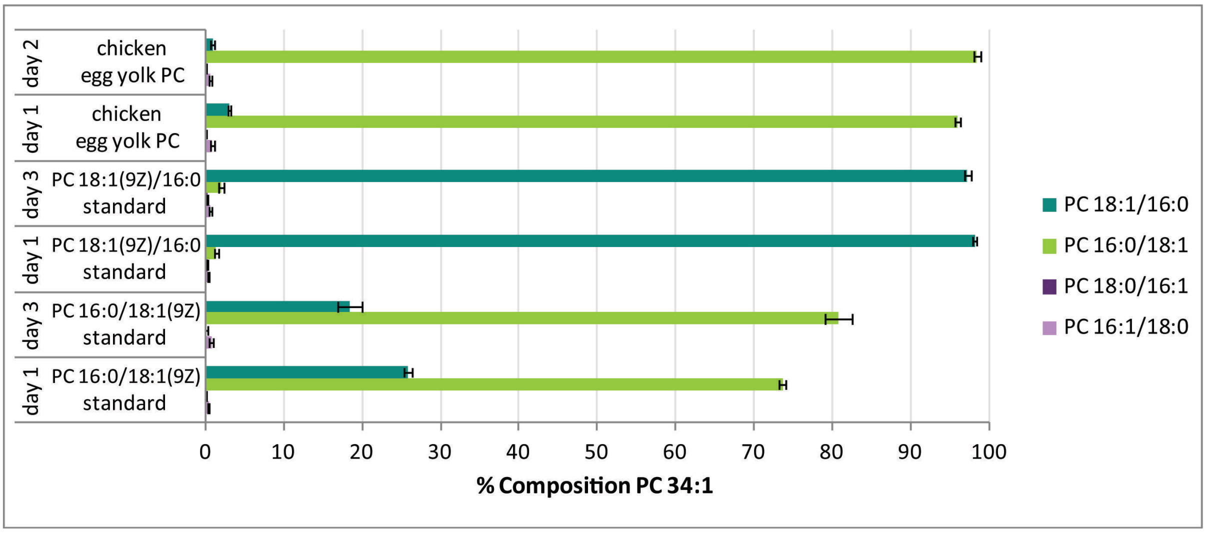

Figure S2. The abundance of *sn*-positional isomers PC 18:1/16:0 and PC 16:0/18:1 as a percentage of phosphatidylcholines of composition PC 34:1 in different biological and synthetic samples. Isomer contributions are estimated from the normalised product ion abundances in DESI-CID/OzID mass spectra as described in the text. The inter-day comparison of composition of *sn*-positional isomers of PC 34:1 present in synthetic standards, PC 16:0/18:1 and PC 18:1/16:0 and an egg yolk phosphatidylcholine extract is shown. All data points represent an average of at least four technical replicates. Uncertainties shown represent standard deviation. Spectra acquired using offline ozone generation (see Methods).

Comparison of the relative rates of reaction of lipid isomers with ambient ozone

There are several opportunities during sample preparation and analysis for unsaturated lipids to be exposed to ambient ozone present in laboratory air. Previous studies have shown this reaction can be rapid.[5,6] It was thus necessary to establish whether the *sn*-positional isomers would have similar or different rates of reaction under these conditions as this could alter the relative populations of isomers in the sample arrays.

To investigate this effect the synthetic PC isomers PC 16:0/18:1(9*Z*) and PC 18:1(9*Z*)/16:0 were deposited onto PTFE spots in a sample array and were left on the bench for 15, 180 or 1440 mins. Following these intervals the arrays were analyzed using DESI-MS in positive ion mode (as described in the Methods section). The abundances of ions at *m/z* 830 corresponding to the lipid ozonide ions, $[M+Na+O_3]^+$, were compared to the abundances of the remaining un-reacted lipid at *m/z* 782. Under these conditions formation of ozonides on the surface was found to be minimal after an exposure of 15 mins. Given the procedures for sample preparation and storage used in this study (see Methods) this suggest a negligible effect on any results reported herein.

The data acquired at longer exposure times are summarized in Figure S4 and show the significant production of ozonides; consistent with prior reports. Importantly however, the ozonide production is comparable for the two isomers investigated suggesting that ambient ozonolysis will have a negligible impact of composition estimates based on DESI-CID/OzID analysis.

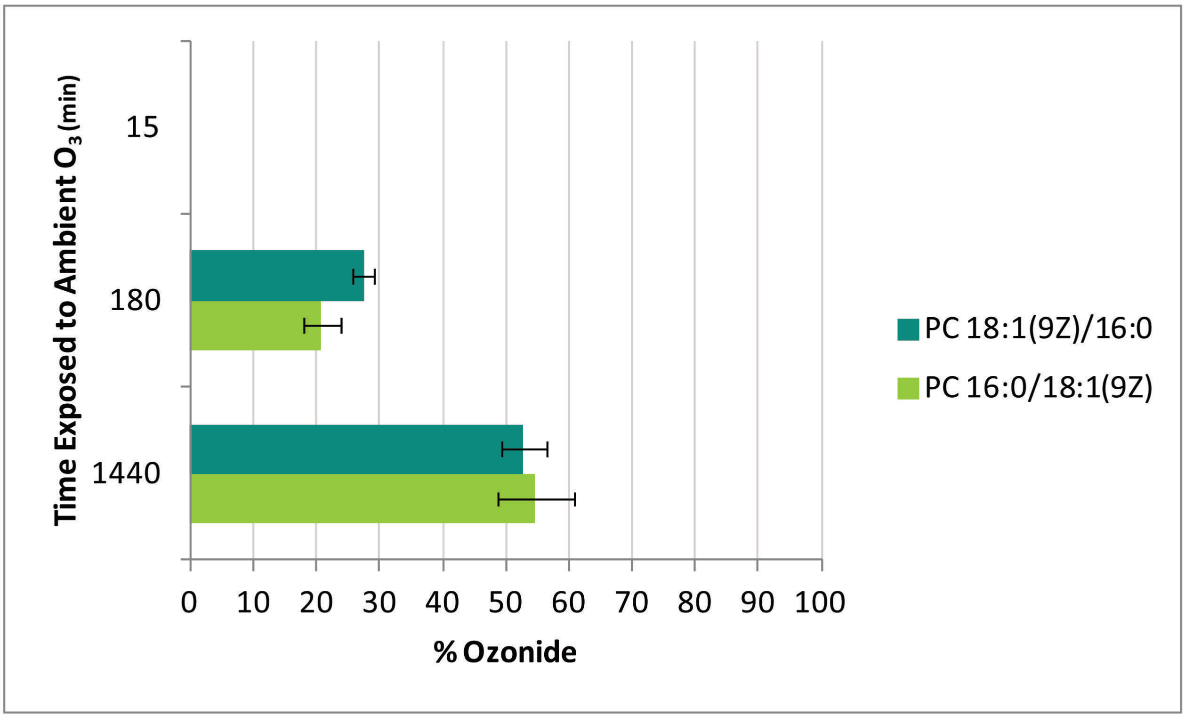

Figure S3. The abundance of ozonides formed from the reaction of unsaturated lipids deposited on PTFE spots with ambient ozone present in laboratory air. Under positive ion DESI-MS analysis, ozonides were detected as $[M+Na+O_3]^+$ ions (*m/z* 830) and are shown as a percentage of the synthetic standards PC 18:1/16:0 or PC 16:0/18:1 detected as $[M+Na]^+$ ions (*m/z* 782). All data points represent an average of at least four technical replicates. Uncertainties shown represent the standard deviation.

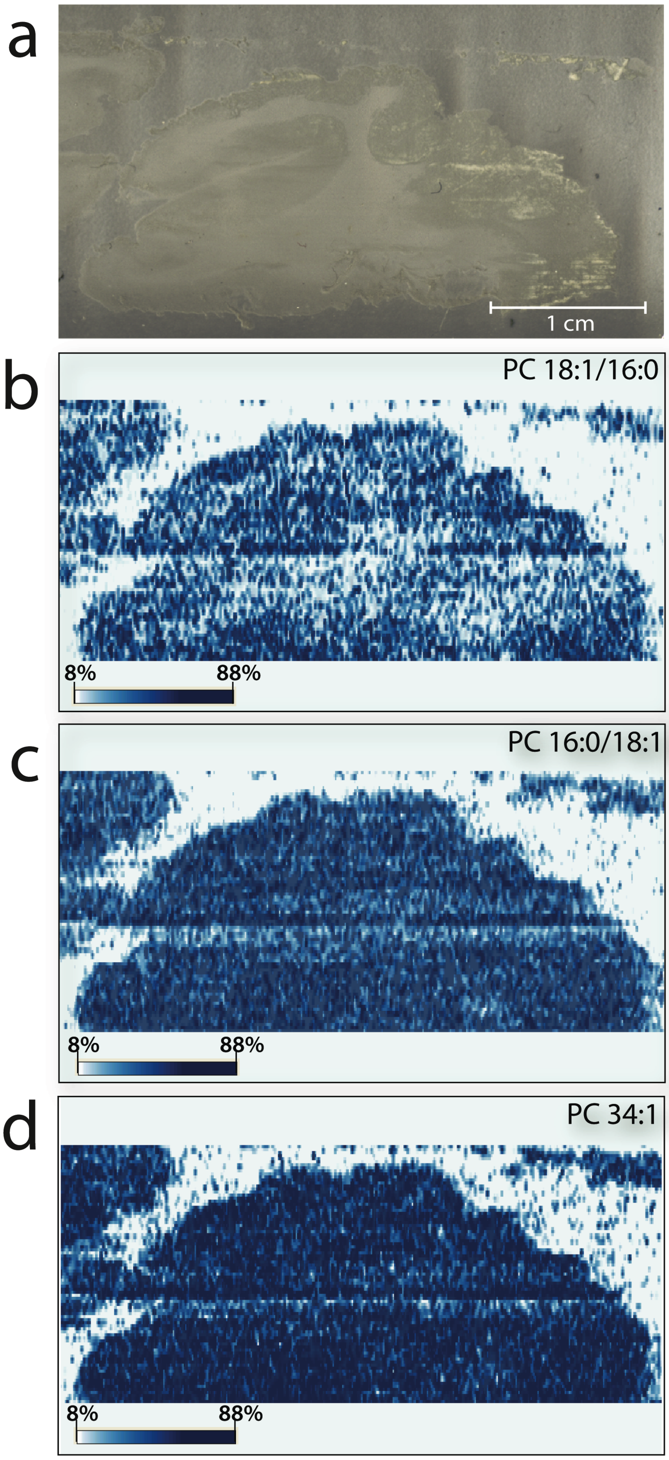

Figure S4. A coronal section of the temporal lobe of the left hemisphere of a sheep brain was dissected and sliced to a thickness of 30 μm as described in the Methods. A small section of the slice was prepared for mounting onto a glass slide and was positioned with the sagittal face upwards for analysis and the outermost side of the left side of the brain was oriented at the top of the slide. (a) Photographic image of sheep brain section used for direct tissue analysis. Images reconstructed from DESI-MS analysis of the tissue. The abundance of diagnostic product ions observed in the DESI-CID/OzID (782 → 599 →) spectra acquired at each position are illustrated for (b) *m/z* 405 and 421 indicative of the local abundance of PC 18:1/16:0; (c) *m/z* 379 and 395 indicative of the local abundance of PC 16:0/18:1; and (d) and *m/z* 599 and 615. All reconstructed ion images are normalized with the most abundant product ion signal detected as 100% and the intensity scale

indicated by the darkness of the blue color is shown under each image for all panels. Note that, the line across the sample image is due to a software communication error; the sample analysis was restarted from this point

			sheep						cow				chicken	synthetic standards				
			liver	kidney		brain		heart	liver	kidney		eye	egg	PC 36:1		PC 36:2	PC 34:1	
			left lobe	medulla	perinephric fat	grey matter	white matter	ventricle	left lobe	medulla	perinephric fat	lens core	yolk	PC 18:0/ 18:1(9Z)	PC 18:1(9Z)/ 18:0	PC 18:1(9Z)/ 18:1(9Z)	PC 16:0/ 18:1(9Z)	PC 18:1(9Z)/ 16:0
**PC 36:1 (m/z 810)**	PC 16:0/20:1	average	0.2	0.3	0.9	1.4	4.4	1.1	0.3	0.4	1.1	3.7	0.6	0.3	0.0			
	(m/z 379, 395)	+/-	0.3	0.2	1.1	1.2	1.7	1.4	0.2	0.1	1.1	4.8	0.3	0.1	0.0			
	PC 20:1/16:0	average	0.1	0.2	1.1	5.6	2.4	0.5	0.2	0.8	0.1	39.6	0.0	0.0	0.0			
	(m/z 433, 449)	+/-	0.3	0.2	1.3	2.2	2.0	3.8	0.2	0.5	0.3	25.4	0.1	0.0	0.0			
	PC 18:0/18:1	average	95.1	88.9	90.8	78.3	73.8	90.1	97.1	78.5	95.0	53.6	94.2	87.7	6.1			
	(m/z 407, 423)	+/-	2.4	3.4	3.7	5.9	8.6	7.2	0.5	1.6	1.2	28.0	1.7	0.1	0.1			
	PC 18:1/18:0	average	4.7	10.6	7.3	14.7	19.5	8.4	2.5	20.3	3.8	3.1	5.2	12.0	93.9			
	(m/z 405, 421)	+/-	2.1	3.3	2.9	6.8	9.0	5.4	0.5	2.0	1.6	4.7	1.6	0.2	0.2			
**PC 36:2 (m/z 808)**	PC 18:0/18:2	average	59.2	26.5	47.5	4.2	1.7	51.5	75.3	25.4	22.9	41.2	53.5			0.1		
	(m/z 407, 423)	+/-	13.6	3.9	15.4	2.9	1.6	10.8	3.1	0.3	6.1	23.8	4.6			0.1		
	PC 18:2/18:0	average	1.7	1.7	1.9	0.6	0.9	3.7	1.6	2.0	0.0	4.1	1.8			0.4		
	(m/z 403, 419)	+/-	0.8	1.0	2.2	0.7	0.8	1.1	0.8	0.4	0.0	9.2	0.7			0.1		
	PC 18:1/18:1	average	39.1	71.8	50.6	95.2	97.4	44.8	23.2	72.6	77.1	54.7	44.7			99.8		
	(m/z 405, 421)	+/-	14.3	3.9	15.9	2.8	1.9	10.3	2.8	0.6	6.1	25.9	4.9			0.1		
**PC 34:1 (m/z 782)**	PC 16:0/18:1	average	96.8	93.4	81.9	60.3	83.9	94.9	95.5	82.3	96.4	39.1	97.2				79.28	1.85
	(m/z 379, 395)	+/-	1.0	1.3	11.4	3.4	4.7	1.1	0.8	0.5	1.1	7.5	1.4				3.35	0.43
	PC 18:1/16:0	average	2.4	5.7	16.6	38.8	15.3	4.3	3.1	16.7	2.2	59.4	2.0				19.96	97.42
	(m/z 405, 421)	+/-	0.9	1.2	11.0	3.4	4.8	1.1	0.7	0.4	0.8	7.1	1.2				3.42	0.54
	PC 16:1/18:0	average	0.6	0.6	0.9	0.5	0.6	0.6	0.8	0.8	0.7	0.9	0.7				0.65	0.52
	(m/z 377, 393)	+/-	0.3	0.3	0.6	0.5	0.6	0.2	0.2	0.1	0.2	0.9	0.2				0.23	0.21
	PC 18:0/16:1	average	0.2	0.2	0.6	0.3	0.2	0.3	0.6	0.3	0.8	0.6	0.1				0.11	0.21
	(m/z 407, 423)	+/-	0.2	0.1	0.4	0.2	0.2	0.2	0.1	0.1	0.3	1.0	0.1				0.16	0.10
**PC 34:2 (m/z 780)**	PC 16:0/18:2	average											85.4					
	(m/z 379, 395)	+/-											1.5					
	PC 18:2/16:0	average											2.0					
	(m/z 403, 419)	+/-											0.7					
	PC 16:1/18:1	average											12.3					
	(m/z 377, 393)	+/-											1.8					
	PC 18:1/16:1	average											0.2					
	(m/z 405, 421)	+/-											0.1					

Table S1. Percent Composition for phosphatidylcholine *sn*-positional isomers. Comparison of abundance of PC 36:1, PC 36:2 and PC 34:1 *sn*-positional isomers in biological tissues using DESI CID/OzID. The composition of PC *sn*-positional isomers present in synthetic standards are also included for comparison with tissue extracts. The composition of each *sn*-positional isomer as a % of total PC 36:1, 36:2 or 34:1 respectively for each tissue extract is shown ("average"). The standard deviation* ("+/-") is shown directly below each corresponding average.

*To calculate the standard deviation, different types of replicate measurements were used. For all samples, a minimum of 4 sample spot replicates were analyzed. Biological replicates were as follows: 5 x sheep brain white matter of temporal lobe, 5 x sheep brain grey matter of temporal lobe, 3 x left lobe of sheep liver, 3 x medulla of sheep kidney, 3 x perinephric fat of sheep kidney, 3 x sheep heart ventricle, 6 x cow eye lens core (m/z 782 only, otherwise 1 biological replicate used), 1 x left lobe of cow liver, 1 x medulla of cow kidney and 1 x perinephric fat of cow kidney. Sheep brain extracts, cow ocular lens extracts and synthetic standards, PC 16:0/18:1(9Z) and PC 18:1(9Z)/16:0 were analyzed on 2 separate days.

Table S2. Comparing the purity of commercially available synthetic phosphatidylcholines PC 16:0/18:1 and PC 18:1/16:0 measured in this study with other reports using alternative mass spectrometric methods. It should be noted that batch-to-batch variation in synthetic preparations may also give rise to some variation in these measurements.

**Reference**	**Citation**	**Method**	**% Alternate isomer (*i.e.*, synthetic "impurity") in PC 16:0/18:1**	**% Alternate isomer (*i.e.*, synthetic "impurity") in PC 18:1/16:0**
This study	Kozlowski *et al.* **2015**	DESI-CID/OzID	20%	2%
Ref [7]	Maccarone *et al. J. Lipid Res.* **2014**, *55*, 1668-1677	Ion-mobility separation	13%	1%
Ref [7]	Maccarone *et al. J. Lipid Res.* **2014**, *55*, 1668-1677	PLA_2 enzyme assay	13%	3%
Ref [7]	Maccarone *et al. J. Lipid Res.* **2014**, *55*, 1668-1677	CID/OzID	17%	4%
Ref [8]	Pham *et al. Analyst* **2014**, *139*, 204-214	CID/OzID	17%	3%
Ref [8]	Pham *et al. Analyst* **2014**, *139*, 204-214	CID (+ve and -ve ion modes)	21-23%	10-16%
Ref [9]	Ekroos *et al.* **2003**, *44*, 2181-2192.	CID (-ve ion modes)	17%	7%

References – Supporting Information

1 Thomas, M. C., Mitchell, T. W. & Blanksby, S. J. OnLine ozonolysis methods for the determination of double bond position in unsaturated lipids. *Methods Mol. Biol.* **579**, 413-441 (2009).

2 Poad, B. L. *et al.* Ozone-induced dissociation on a modified tandem linear ion-trap: observations of different reactivity for isomeric lipids. *J. Am. Soc. Mass. Spectrom.* **21**, 1989-1999 (2010).

3 Pham, H. T. *et al.* Structural characterization of glycerophospholipids by combinations of ozone- and collision-induced dissociation mass spectrometry: the next step towards "top-down" lipidomics. *Analyst* **139**, 204-214 (2014).

4 Pham, H. T., Maccarone, A. T., Campbell, J. L., Mitchell, T. W. & Blanksby, S. J. Ozone-induced dissociation of conjugated lipids reveals significant reaction rate enhancements and characteristic odd-electron product ions. *J. Am. Soc. Mass. Spectrom.* **24**, 286-296 (2013).

5 Cohen, S. L. Ozone in ambient air as a source of adventitious oxidation. A mass spectrometric study. *Anal. Chem.* **78**, 4352-4362 (2006).

6 Ellis, S. R., Hughes, J. R., Mitchell, T. W., in het Panhuis, M. & Blanksby, S. J. Using ambient ozone for assignment of double bond position in unsaturated lipids. *Analyst* **137**, 1100-1110 (2012).

7 Maccarone, A. T. *et al.* Characterization of acyl chain position in unsaturated phosphatidylcholines using differential mobility-mass spectrometry. *J Lipid Res* **55**, 1668-1677 (2014).

8 Pham, H. T. *et al.* Structural characterization of glycerophospholipids by combinations of ozone- and collision-induced dissociation mass spectrometry: The next step towards "top-down" lipidomics. *Analyst* **139**, 204-214 (2014).

9 Ekroos, K. *et al.* Charting molecular composition of phosphatidylcholines by fatty acid scanning and ion trap MS3 fragmentation. *J Lipid Res* **44**, 2181-2192 (2003).
